# Descriptive Characteristics of Surface Water Quality in Hong Kong by a Self-Organising Map

**DOI:** 10.3390/ijerph13010115

**Published:** 2016-01-08

**Authors:** Yan An, Zhihong Zou, Ranran Li

**Affiliations:** School of Economics and Management, Beihang University, Beijing 100191, China; anyanmog@163.com (Y.A.); liranran1101@163.com (R.L.)

**Keywords:** water quality, self-organising map, principal component analysis

## Abstract

In this study, principal component analysis (PCA) and a self-organising map (SOM) were used to analyse a complex dataset obtained from the river water monitoring stations in the Tolo Harbor and Channel Water Control Zone (Hong Kong), covering the period of 2009–2011. PCA was initially applied to identify the principal components (PCs) among the nonlinear and complex surface water quality parameters. SOM followed PCA, and was implemented to analyze the complex relationships and behaviors of the parameters. The results reveal that PCA reduced the multidimensional parameters to four significant PCs which are combinations of the original ones. The positive and inverse relationships of the parameters were shown explicitly by pattern analysis in the component planes. It was found that PCA and SOM are efficient tools to capture and analyze the behavior of multivariable, complex, and nonlinear related surface water quality data.

## 1. Introduction

Water quality is assuming great importance with rising pressure on industry, agriculture, and population [1]. Surface water is a substantial source for domestic usage, industrial heating, and agricultural irrigation. Since surface water is easily accessible to human beings, it is the most vulnerable water body to contaminants. The major pollutant sources of surface water include discharges from domestic, industrial and agricultural activities. Runoff transports pollutants in urban areas and agricultural lands to surface water resources, such as rivers and lakes [2]. Due to the complexity and uncertainty involved in surface water interaction mechanism into groundwater, pollutants in surface water appear significant for groundwater quality. Therefore, the assessment of surface water quality is of great concern in water environmental management.

Water quality monitoring is time-consuming and it is expensive to obtain a large number of water quality data. The water quality data analysis seems a difficult task because the data are multidimensional, complex, and nonlinear. There are a variety of methods to assess water quality, such as TOPSIS method [3,4,5], statistical analysis [6,7,8], support vector machine [9], set pair analysis [10], water quality index [11], and matter-element analysis [12]. However, it is difficult to decide which ones are better. Owing to the complex characteristics of surface water, it is necessary to use a sophisticated knowledge extraction and diagnosis tool that can provide the analysis and visualization of the multidimensional data set [13]. Model-based diagnosis technique and statistics-based diagnosis technique are widely used to solve the problem. However, the model-based diagnosis technique has some weaknesses. These weaknesses include: (1) they cannot provide an entirely satisfactory description of the cause-effect relationships; (2) the models require the specification of a large number of parameters; and (3) a large number of parameters included in this model need to respecify parameter values for different operational conditions [14]. Conversely, statistics-based diagnosis techniques are preferable for implementing knowledge extraction in water quality data. Multivariate analysis methods, such as principal component analysis (PCA), belong to a kind of statistics-based diagnosis technique and have been widely developed in hydrological system analysis [15,16,17,18]. However, the limitations of classical multivariate analysis methods are well known [2,19]. Artificial neural network (ANN) is another type of statistics-based diagnosis technique and powerful for multivariate, nonlinear analysis. ANN offers an alternative to traditional statistical methods for optimal monitoring and determination of dynamic system [20], and has attracted considerable attention [21,22].

Self-organising map (SOM) is a neural network-based pattern analysis technique with unsupervised learning [23,24], that has been widely applied in water quality data analysis [2,11,25,26,27]. Çinar and Merdun employed SOM to diagnose the relationships of surface water quality parameters, and clustered seven groups corresponding to water quality parameters [2]. Hong and Rosen used the SOM technique to capture the influences of stormwater infiltration on groundwater quality parameters, and obtained the relationships between the parameters [11]. Wu *et al.* performed a SOM method to identify the effects caused by climate change and human activities on coastal water quality [26]. The SOM technique is a powerful tool to group the similar input patterns from a multidimensional input space into a much lower dimensional space, usually two dimensions. SOM can be used for clustering, classification, estimation, prediction, and data mining [28]. SOM can potentially outperform current methods of analysis because they can successfully: (1) deal with the nonlinearities of the system; (2) be developed from data without requiring the mechanistic knowledge of the system; (3) handle noisy or irregular data; (4) be easily and quickly updated; and (5) interpret information from multiple variables or parameters [13,14]. The SOM method has excellent visualization capabilities, which can be helpful in the initial steps of water quality assessment frameworks.

In this study, PCA was performed to extract four significant principal components (PCs) from the twelve water quality parameters, and the SOM method has been used to analyze the complex relationships of water quality parameters in multivariable surface water quality data.

## 2. Study Area and Data

**Figure 1:**
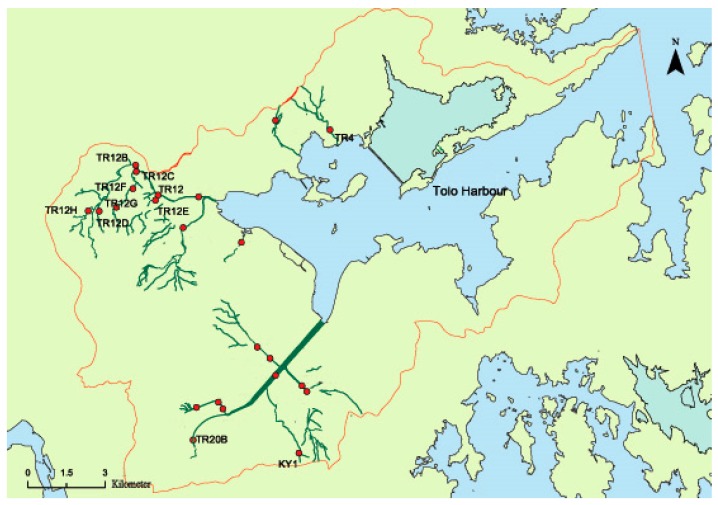
Locations of the 11 monitoring stations in the zone.

Hong Kong is divided into ten water control zones and each one has a set of water quality objectives. The rates of annual compliance with the key water quality objectives are assessed during the year. The Tolo Harbor and Channel Water Control Zone is one of the ten water control zones in Hong Kong. Tolo Harbor is largely landlocked, with a narrow channel to the open sea, making it difficult for pollutants entering the harbor to be flushed out by tidal action. The harbor suffered severely from red tides in the 1980s. The establishment of the zone aimed to help improve the harbor water quality as well. The rivers in the zone are all short, with relatively small flows. They are easily affected by the rainfall and runoff. The zone includes 23 monitoring stations across 10 watercourses: Kwun Yam Shan Stream, Lam Tsuen River, Shan Liu Stream, Shing Mun River, Siu Lek Yuen Nu llah, Tai Po Kau Stream, Tai Po River, Tai Wai Nullah, Tin Sum Nullah, and Tung Tze Stream. Some watercourses are easily affected by coastal water. Therefore, four watercourses (Kwun Yam Shan Stream, Lam Tsuen River, Shan Liu Stream, and Tin Sum Nullah) were selected as research targets. Kwun Yam Shan Stream and Tin Sun Nullah are the tributary streams of Shing Mun River. Shing Mun River, Lam Tsuen River, and Shan Liu Stream empty into Tolo harbor. The eleven monitoring stations (KY1, TR12, TR12B, TR12C, TR12D, TR12E, TR12F, TR12G, TR12H, TR4, TR20B) of the four watercourses were used in this study (Figure 1). KY1, TR4, and TR20B are located in Kwun Yam Shan Stream, Shan Liu Stream, and Tin Sum Nullah, respectively. The other eight monitoring stations are situated in Lam Tsuen River. The locations of the eleven monitoring stations are shown in Figure 1, which is obtained from the website of Hong Kong Environmental Protection Department (HKEPD). The red spots in Figure 1 are all the 23 monitoring stations in the study area.

The water quality data were collected for the twelve parameters during 2009–2011 with a total of 4752 measurements [29]. The water quality parameters involved were 5-day biological oxygen demand (BOD_5_), ammonia- nitrogen (NH_3_-N), chemical oxygen demand (COD), electrical conductivity (EC), dissolved oxygen (DO), total phosphorus (TP), nitrate nitrogen (NO_3_-N), nitrite nitrogen (NO_2_-N), saturated oxygen (Satur O_2_), non-dissolved matter (Susp), dissolved matter (Diss sol), and temperature (T).

## 3. Methodology

### 3.1. Principal Component Analysis

PCA is an efficient tool to explain the variance of a large data set of correlated parameters with a much smaller data set of uncorrelated PCs [30,31]. The PCs acquired by multiplying the original correlated parameters with the eigenvector (loadings), can provide information on the most meaningful parameters that describe a whole data set allowing data reduction with minimum loss of original information [32,33].

### 3.2. Self-Organising Map

**Figure 2:**
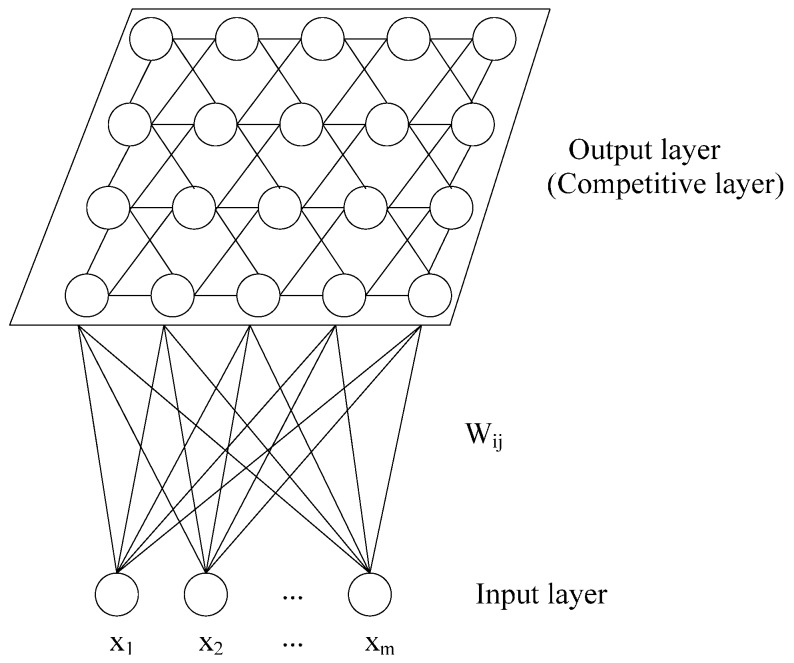
Topological structure of SOM.

SOM has been extensively used for data analysis owing to its excellent ability for displaying a high-dimensional dataset into a lower dimensional space. SOM consists of input layer and output layer (competitive layer), connected with each other by computational weights. The input layer is connected to each vector of the data set, and the output layer is made of an array of nodes (Figure 2).

The neurons in a SOM learn in an unsupervised way because the network is not required to provide a specific objective. SOMs are competitive networks so that the neurons compete to provide the right answer, with only one neuron (or one node of neurons) becoming activated when a data pattern is presented [34]. It involves the processes of competition, cooperation, and update. The best-matching unit (BMU) is decided in the process of competition, the neighbor neurons are determined in the process of cooperation, and the weight vectors are updated in the last process. The steps of SOM algorithm are displayed as follows:

(1) Initialize the SOM network. The weight vector *w_ij_* (*i* = 1,2,…,*S*; *j*=1,2,…,*R*) is set randomly in the interval [0,1], *R* is the sample dimension, and *S* is the number of output neurons. The initial value of learning ratio η(0) (0 < η(*t*) < 1), the map size, the neighborhood ratio *N_g_*(0), and the maximum number or possible iterations T are defined.

(2) Present an input vector $P_{k}=\left(p_{1}^{k},p_{2}^{k},\cdots p_{R}^{k}\right)$ (k = 1,2,…,*M*, *M* is the sample number) to the SOM network and calculate the distance. The Euclidean distance is frequently used and can be calculated as:

(1) $$d_{i}=\sqrt{\sum_{j=1}^{R}{\left(p_{j}^{k}-w_{ij}\right)}^{2}},i=1,2,\cdots ,S.$$

(3) Choose the smallest distance and identify the BMU.

(4) Update the weight vector *w_ij_* in the neighbor ratio *N_g_*(*t*).

(2) $$w_{ij}\left(t+1\right)=w_{ij}\left(t\right)+\eta \left(t\right)\left(p_{j}^{k}-w_{ij}\left(t\right)\right),$$

where $w_{ij}(t+1)$ is the weight vector at learning step t + 1. The neighbor ratio *N_g_*(*t*) and the learning ratio η(t) decrease with the number of iterations of the model.

(5) The process goes on an iterative way until the optimal number of iteration steps is satisfied and then it jumps back to step (2).

In step (1), the map size is vital to detect the deviation of the data set. If the map size is too small, it might not explain some important differences that should be detected. In contrast, if the map size is too big, the differences are too small [26,35]. There are two classical methods to determine the map size [26,36]. The first method is to calculate quantization error (QE) and topographic error (TE), and the other one is that the optimal number of neurons is close to $5\sqrt{n}$ (*n* is the number of samples of the training data). The detailed discussion of QE and TE can be seen in [36,37].

The SOM technique has a distinct capability to represent the complex relationships of the water quality parameters using component planes and U-matrix. All simulations were implemented in MATLAB R2012b using a SOM toolbox [38].

### 3.3. Data Pre-Processing

The water quality parameters usually have different units and need to be normalized before applying the SOM method to avoid misclassification. The data can be transformed to the data with zero mean and unit variance.

### 3.4. K-Means Clustering

K-means clustering is a method of vector quantization, aiming to partition n observations into *k* (k≤n) sets $S=\{S_{1},S_{2},\cdots ,S_{k}\}$ to minimize the within-cluster sum of squares:

(3) $$E=\sum_{i=1}^{k}\sum_{x_{i}\in S_{i}}||x_{i}-\mu_{i}|{|}^{2},$$

where $\left(x_{1},x_{2},\cdots ,x_{n}\right)$ is a set of observations, k is the number of clusters, $\mu_{i}$ is the mean of points in $S_{i}$. The Davies-Bouldin clustering index was used to determine the optimal number of the clusters for a dataset, and k-means clustering was conducted in MATLAB R2012b.

## 4. Results and Discussion

### 4.1. Statistical Analysis

The statistical characteristics and Pearson correlation matrix of the twelve water quality parameters are listed in Table 1 and Table 2, respectively. Descriptive statistics includes minimum values, maximum values, median values, mean values, standard deviation (SD), and coefficient of variation (CV) of the water quality data in Table 1. Table 2 reveals the quantitative representation of these relationships for the parameters. As it can be seen in Table 1, Susp has the biggest CV, followed by NH_3_-N, while Satur O_2_ has the smallest, followed by DO. This demonstrates that Susp and NH_3_-N change a lot, while Satur O_2_ and DO are temporally stable. Except for Susp, NH_3_-N, Satur O_2_, and DO, the other parameters possess medium CVs, which indicates their concentrations do not change as much as Susp and NH_3_-N, but more than Satur O_2_ and DO.

**Table 1:** Statistical description of water quality parameters across the sample points.

Water Quality Parameter	Unit	Minimum	Maximum	Median	Mean	SD	CV
BOD_5_	mg·L^−1^	0.01	17	0.8	1.6716	2.3262	1.3916
NH_3_-N	mg·L^−1^	0.006	13	0.056	0.6021	1.7633	2.9285
COD	mg·L^−1^	0.3	46	4	5.2942	4.4706	0.8444
EC	μS/cm	29	2018	157	206.5278	184.1479	0.8916
DO	mg·L^−1^	4.3	10.6	8.1	8.1083	1.1248	0.1387
TP	mg·L^−1^	0.005	1.4	0.09	0.1674	0.2348	1.4030
NO_3_-N	mg·L^−1^	0.026	4.6	0.81	1.0202	0.7751	0.7598
NO_2_-N	mg·L^−1^	0.00007	1.5	0.007	0.0674	0.1735	2.5731
Satur O_2_	%	48	130	98	94.9672	11.5266	0.1214
Susp	mg·L^−1^	0.1	650	3.4	9.9902	37.1404	3.7177
Diss sol	mg·L^−1^	27.1	1296.3	99	112.0402	95.6936	0.8541
T	°C	11.2	33.4	23.5	23.4907	4.3935	0.1870

T is one of the most important water quality parameter in water quality, and limits the saturation values of gases and solids that are dissolved in it [39]. T varies between 11.2 °C and 33.4 °C with a median value of 23.5 °C. T shows negative correlation with DO (*r* = −0.466) and positive correlation with BOD_5_, NH_3_-N, COD, EC, TP, NO_3_-N, NO_2_-N, Satur O_2_, Susp, and Diss sol, which are presented in Table 2.

**Table 2:** Pearson correlation matrix for the water quality parameters across the sample points.

Water Quality Parameter	BOD_5_	NH_3_-N	COD	EC	DO	TP	NO_3_-N	NO_2_-N	Satur O_2_	Susp	Diss Sol	T
BOD_5_	1.000											
NH_3_-N	0.646 **	1.000										
COD	0.667 **	0.741 **	1.000									
EC	0.232 **	0.214 **	0.236 **	1.000								
DO	−0.340 **	−0.182 **	−0.350 **	−0.315 **	1.000							
TP	0.694 **	0.872 **	0.795 **	0.355 **	−0.274 **	1.000						
NO_3_-N	0.312 **	0.130 **	0.263 **	0.416 **	−0.355 **	0.398 **	1.000					
NO_2_-N	0.451 **	0.406 **	0.442 **	0.318 **	−0.043	0.646 **	0.471 **	1.000				
Satur O_2_	−0.352 **	−0.159 **	−0.300 **	−0.242 **	0.784 **	−0.234 **	−0.372 **	0.086	1.000			
Susp	0.256 **	0.059	0.198 **	0.015	−0.103 *	0.132 **	0.063	0.040	−0.077	1.000		
Diss sol	0.277 **	0.278 **	0.343 **	0.705 **	−0.060	0.426 **	0.365 **	0.516 **	0.047	0.059	1.000	
T	0.027	0.051	0.111 *	0.156 **	−0.466 **	0.080	0.015	0.167 **	0.177 **	0.052	0.151 **	1.000

Notes: ** indicates correlation is significant at the 0.01 level (2-tailed); * indicates correlation is significant at the 0.05 level (2-tailed).

The concentration of oxygen in surface water is a measure of self-cleaning capacity of the water body. 14 out of the 396 samples of DO concentration are below 6 mg·L^−1^, and the median value and mean value of the DO concentration are 8.1 mg·L^−1^ and 8.1083 mg·L^−1^, respectively. Similarly, the median value and mean value of Satur O_2_ are 98% and 94.9672%, respectively. DO and Satur O_2_ are negatively correlated with BOD_5_, NH_3_-N, COD, EC, TP, NO_3_-N, and Susp. DO exhibits a strong positive correlation with Satur O_2_ with a correlation coefficient of 0.784.

EC varies in the range of 29 μS/cm and 2018 μS/cm with a median value of 157 μS/cm. The maximum value of 2018 μS/cm occurred with Diss sol concentration as 1296.3 mg·L^−1^ (the maximum value of Diss sol concentration). EC is positively correlated with Diss sol, with a correlation coefficient of 0.705, which is expressed in Table 2. It means that an increase in Diss sol leads to an increase in EC. BOD_5_, NH_3_-N, COD, and TP have relatively good positive relationships with each other. BOD_5_ is positively correlated with NH_3_-N, COD and TP, with correlation coefficients of 0.646, 0.667 and 0.694, respectively. NH_3_-N has positive relationships with COD and TP, with correlation coefficients of 0.741 and 0.872, respectively. TP and COD are correlated with a correlation coefficient of 0.795. It is noted the NO_2_-N has relatively good positive relationship with TP (0.646), and slightly positive relationships with BOD_5_ (0.451), NH_3_-N (0.406), and COD (0.442).

### 4.2. PCA Results

The KMO test and Bartlett’s test were firstly implemented to examine the validity of PCA (Table 3). The test shows that the KMO and Bartlett’s test are 0.626 and 4517.867, respectively. It means that PCA can be used to perform data reduction.

**Table 3:** KMO and Bartlett’s test.

KMO Measure of Sampling Adequacy	Bartlett’s Test of Sphericity		
	Approx. Chi-Square	df	Sig.
0.626	4517.867	66	0.000

The objective of PCA is to reduce the multidimensional parameters to a set of PCs much smaller in number. According to the criteria of eigenvalue-one, four PCs were extracted, accounting for 75.894% of the total variance (Table 4). The PCs, eigenvalues, percentage of total variance, and cumulative percentage of explained variance are shown in Table 4. 

**Table 4:** Loading on components for water quality parameters.

Water Quality Parameter	PC			
	PC1	PC2	PC3	PC4
BOD_5_	0.782			
NH_3_-N	0.755			
COD	0.819			
EC			0.629	
DO		0.787		
TP	0.901			
NO_3_-N	0.565			
NO_2_-N	0.663			
Satur O_2_		0.825		
Susp				
Diss sol	0.582		0.544	
T				0.886
Eigenvalue	4.582	1.784	1.557	1.184
Percentage of total variance	38.187	14.866	12.978	9.864
Cumulative percentage of variance	38.187	53.053	66.030	75.894

Note: non-significant correlation coefficients are not shown.

As it can be seen in Table 4, the eigenvalues of four PCs are 4.582, 1.784, 1.557, and 1.184, respectively. The four PCs can explain 75.894% of the total variance. The first two PCs (PC1 and PC2) account for 38.187% and 14.866% of the variance, respectively, explaining more than a half of the total variance in the original dataset. PC3 and PC4 explain 12.978% and 9.864% of the total variance, respectively.The first PC (PC1) with the biggest eigenvalue 4.582 has strong positive loadings on TP, COD, BOD_5_, NH_3_-N, NO_2_-N, Diss sol, and NO_3_-N, which suggests that PC1 represents the contaminants in the study area. The coefficients of TP, COD, BOD_5_, NH_3_-N, and NO_2_-N are higher than those of NO_2_-N, Diss sol, and NO_3_-N. It means that TP, COD, BOD_5_, NH_3_-N, and NO_2_-N have bigger effect on PC1 than the other two parameters. PC2 has significant loadings by Satur O_2_ and DO, representing the dissolved oxygen in the water body. PC3 has positive loadings on EC and Diss sol. EC and Diss sol are correlated as mentioned above. The existence of high concentration of Diss sol leads to the high loadings of EC. The last PC (PC4) indicates the temperature because it only has a strong loading by T. Difference in water temperature affects dissolved oxygen, the rate of photosynthesis and metabolic rates of aquatic life [39]. PC2 and PC4 indicate the decay rate of the contaminants. PCA results represent the contaminants and the decay rate of the contaminants regardless of monitoring stations in the study area.

### 4.3. SOM Results

The map size is crucial for SOM technique to cluster the data set. QEs and TEs of big and small map sizes were calculated to determine the optimal number of the map units (Table 5). It can be seen that the map size of (14 × 7) has the minimum values of QE and TE as 1.2388 and 0.0152, respectively. Therefore, SOM involved 98 output neurons displayed in 14 rows and 7 columns is chosen in this study. The total number 98 arranged in the hexagonal grid is close to 99.5 ($5\sqrt{n}$).

**Table 5:** QEs and TEs of different map sizes.

Quality of Trained SOM	Map Size						
	(20 × 10)	(17 × 14)	(15 × 8)	(14 × 7)	(13 × 9)	(10 × 8)	(7 × 6)
QE	7.5403	8.9137	8.8477	**1.2388**	8.0568	7.9560	10.0911
TE	1	0.5429	1	**0.0152**	0.9975	0.9949	1

**Figure 3:**
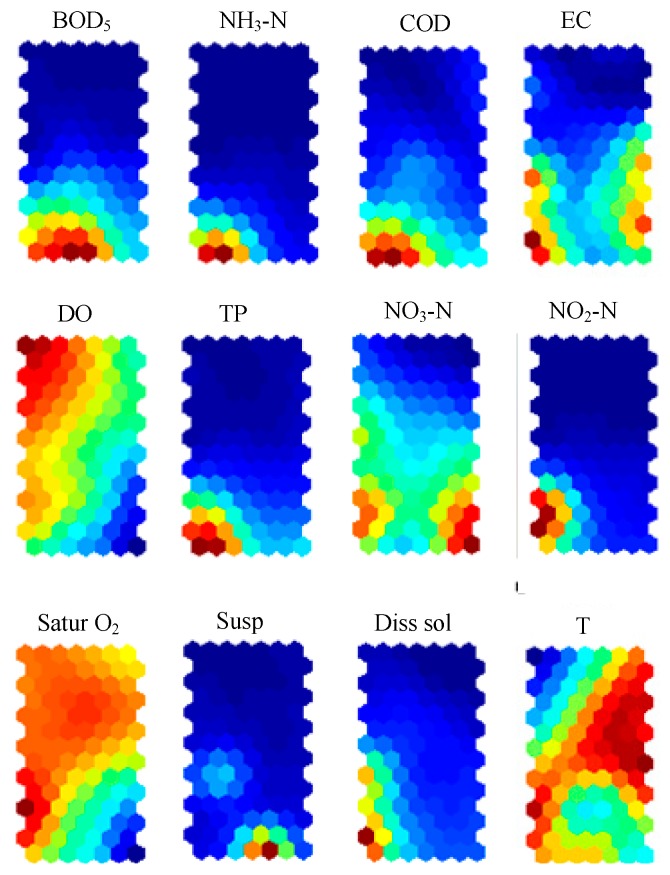
Patterning analysis for the water quality parameters on the SOM plane.

The visualization of the component planes is a good tool to figure out the interrelationship of the different water quality parameters. By comparing the component planes in Figure 3, some parameters demonstrate positive patterns. The grouping of the parameter planes shows three well-defined groups of correlated parameters. The component planes of the same groups have positive relationships between them. The first group includes the parameters of TP, COD, BOD_5_, NH_3_-N, and NO_2_-N. All the water quality parameters in this group have high values (red color) in the lower parts, especially in the lower left parts of the group. It is shown in Section 4.2 above these five parameters have bigger effect on PC1 than the other two parameters (NO_3_-N and Diss sol). The second group includes EC and Diss sol, which is expressed as PC2 in Section 4.2 above. EC is a reflection of Diss sol in water. The third group comprises DO and Satur O_2_. DO is correlated with Satur O_2_ with a correlation coefficient of 0.784 in Pearson correlation matrix, and PC2 shows strong positive loadings on DO and with Satur O_2_. The non-conventional positions of Susp, T, and NO_3_-N could be explained by their ability to describe various complex pollutants and their transformations [15].

**Figure 4:**
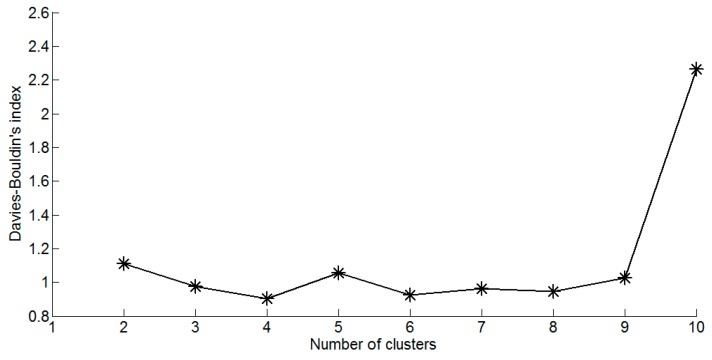
Davies-Bouldin clustering index of the K-means clustering algorithm.

**Figure 5:**
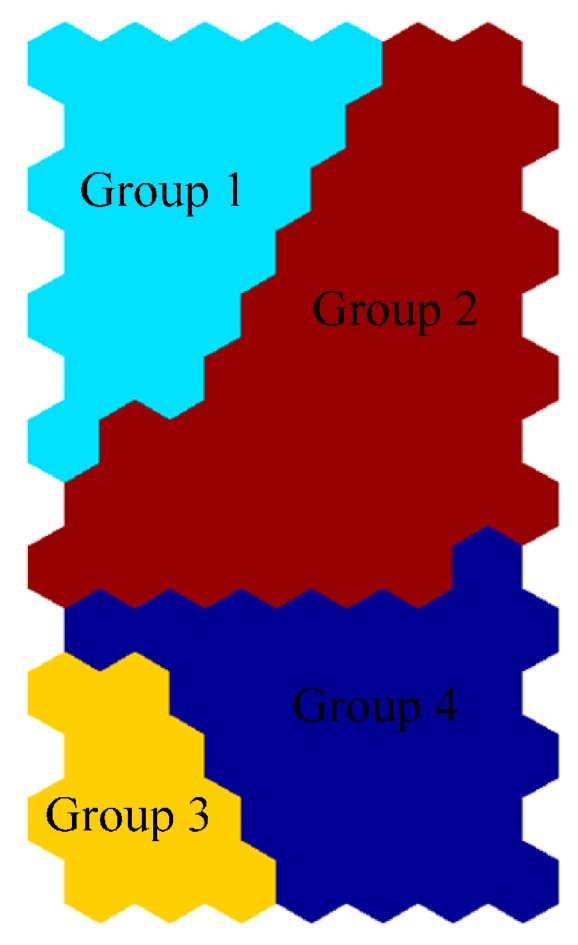
Clusters of the SOM for the water quality dataset.

The cluster analysis of SOM is implemented by K-means clustering algorithm to find the optimal number of the clusters. Davies-Bouldin clustering index [40] is to compute the optimal number of clusters for a dataset, which is commonly used in determining the optimal numbers of clusters [2,14]. As shown in Figure 4, the Davies-Bouldin clustering index is minimized at four with the best clustering. That means the optimal number of the clusters is four, and the four-cluster structure of the map is described in Figure 5. According to Figure 3 and Figure 5, the following information on water quality parameters can be concluded:

(1) High DO, low T, low BOD_5_, low NH_3_-N, low COD, low EC, low TP, low NO_2_-N, low Susp, and low Diss sol (Group 1).

(2) High T, high Satur O_2_, low BOD_5_, low NH_3_-N, low COD, low EC, low TP, low NO_3_-N, low NO_2_-N, low Susp, and low Diss sol (Group 2).

(3) High BOD_5_, high NH_3_-N, high COD, high TP, and low Susp (Group 3).

(4) High NO_3_-N, low NH_3_-N, low TP, low NO_2_-N, and low Diss sol (Group 4).

In winter, when temperature is low, high concentration of DO, low concentrations of BOD_5_, NH_3_-N, COD, EC, TP, NO_2_-N, Susp, and Diss sol are observed in Group 1. Çinar and Merdun clustered seven groups based on 1046 surface water samples collected over six years and found that in winter when temperature is lowest and rainfall highest, high concentration of DO, and low concentrations of Na, K, Cl, NH_4_-N, NO_2_-N, and *o*-PO_4_ (ortho-phosphate), pV (organic matter) can be observed in one group, which is similar to the results in this study [2].

The correlation matrix of the weight of the SOM is shown in Table 6. The minimum values, maximum values, mean values, and SE values of the four groups are expressed in Table 7. As shown in Figure 3 and Figure 5, Groups 2 and 4 represent the normal condition of the study area. The second group contain a total of 199 samples showing the highest frequency among the four groups, followed by the fourth group with a total of 85 samples.

## 5. Conclusions

Water pollution control and management require the interpretation of a large amount of water quality data, and the data are complex, multidimensional, and nonlinearly related. Therefore, the analysis and diagnosis of surface water quality is a quite difficult task due to the characteristic of the data. In this study, PCA and SOM were implemented to discover the complex relationship among the twelve surface water quality parameters during 2009–2011 from river water monitoring stations in Tolo Harbor and Channel Water Control Zone in Hong Kong. The following results were obtained.

(1) In the study area, the overall quality of surface water is good. It is attributable to a number of improvement measures, which had a significant effect on river water quality. However, some monitoring stations were still receiving discharges of untreated sewage effluents in this area. The major contaminants in the study area are TP, COD, BOD_5_, NH_3_-N, and NO_2_-N, which is indicated in Pearson correlation matrix, PCA results, and component planes in SOM. 

(2) Similarly, relationships were observed between Satur O_2_ and DO, EC and Diss sol. It means that Pearson correlation matrix is an efficient tool to analyze the relationships between the parameters, which can verify the relationships in the SOM results.

(3) The raw data can be clustered to four groups, and Groups 2 and 4 describe the normal condition of the study area.

It has been demonstrated that PCA shows good ability in dealing with multivariable data set in this study. Meanwhile, SOM, a powerful artificial intelligence technique, is capable of capturing the complex relationships between the parameters. It is demonstrated in this paper that PCA and SOM are good tools to deal with large data sets, and they may have the potential to be applied solve other types of water resources (groundwater) problems.

**Table 6:** Correlation matrix of the weight of the SOM.

Water Quality Parameter	BOD_5_	NH_3_-N	COD	EC	DO	TP	NO_3_-N	NO_2_-N	Satur O_2_	Susp	Diss Sol	T
BOD_5_	1.000											
NH_3_-N	0.8520 **	1.000										
COD	0.9472 **	0.9452 **	1.000									
EC	0.5582 **	0.5230 **	0.5871 **	1.000								
DO	−0.4076 **	−0.1729	−0.3624 **	−0.5098 **	1.000							
TP	0.8946 **	0.9643 **	0.9636 **	0.6485 **	−0.2656 **	1.000						
NO_3_-N	0.5526 **	0.3704 **	0.5258 **	0.8214 **	−0.5080 **	0.5488 **	1.000					
NO_2_-N	0.6957 **	0.7657 **	0.7645 **	0.6347 **	−0.0349	0.8722 **	0.5593 **	1.000				
Satur O_2_	−0.4349 **	−0.1504	−0.3206 **	−0.4171 **	0.8490 **	−0.2348 **	−0.5284 **	0.0551	1.000			
Susp	0.6325 **	0.2710 **	0.4833 **	0.2591 **	−0.4357 **	0.3537 **	0.4073 **	0.1948	−0.4717 **	1.000		
Diss sol	0.6172 **	0.7041 **	0.7131 **	0.7485 **	−0.0320	0.7879 **	0.6231 **	0.8747 **	0.0843	0.2597 **	1.000	
T	−0.0437	0.0318	0.0730	0.1949	−0.3484 **	0.0452	−0.0294	0.1244	0.1972	−0.0541	0.1700	1.000

Note: ** indicates correlation is significant at the 0.05 level.

**Table 7:** Statistical description of each group.

Water Quality Parameter	Group 1				Group 2				Group 3				Group 4			
	Min	Max	Mean	SE	Min	Max	Mean	SE	Min	Max	Mean	SE	Min	Max	Mean	SE
BOD_5_	0.05	17	3.1412	0.3886	0.01	16	0.9123	0.1118	0.02	2	0.7095	0.1004	0.05	16	2.7442	0.2691
NH_3_-N	0.006	7.7	0.9099	0.1718	0.009	3.1	0.1628	0.0330	0.01	0.99	0.1478	0.0311	0.016	13	1.6104	0.3560
COD	0.8	16	6.6145	0.4327	0.3	22	4.2106	0.2242	0.6	25	4.3907	0.5711	2	46	7.1882	0.7265
EC	148	263	181.6522	2.5830	29	169	92.9548	2.6224	175	307	245.4186	5.6012	282	2018	474.5176	24.4396
DO	4.3	10.1	7.8319	0.1345	4.3	10.5	8.5518	0.0673	6.3	10.6	7.9581	0.1489	5	9.9	7.3894	0.1236
TP	0.01	0.98	0.2431	0.0290	0.005	0.73	0.0795	0.0072	0.03	0.72	0.1242	0.0160	0.03	1.4	0.3326	0.0393
NO_3_-N	0.47	4.6	1.5046	0.1085	0.026	2.6	0.6816	0.0364	0.11	1.8	0.8033	0.0797	0.14	3.6	1.5276	0.0882
NO_2_-N	0.0005	0.8	0.1354	0.0239	0.00007	1.5	0.0211	0.0077	0.0001	0.08	0.0212	0.0043	0.0002	1.3	0.1443	0.0277
Satur O_2_	48	130	93.2174	1.7212	48	117	98.8995	0.5259	72	109	93.4884	1.1433	60	124	87.9765	1.5783
Susp	0.6	650	26.5493	10.1026	0.1	120	5.6302	0.9204	0.6	19	4.1698	0.5063	1.9	41	9.5694	1.0578
Diss sol	102.4	185	135.6246	2.6220	35.8	195	83.5106	1.6858	47.8	148.4	77.5977	4.0243	27.1	1296.3	176.9012	19.9310
T	16.5	33.2	24.0783	0.4486	11.2	29.9	22.9070	0.3121	11.9	29.8	23.7256	0.7340	15.1	33.4	24.1788	0.5001
